# Factors influencing childbearing intention among married childless female nurses in Korea: a cross-sectional study using quantile regression

**DOI:** 10.4069/whn.2026.03.03

**Published:** 2026-03-31

**Authors:** Yoonjoo Jung, Moonjeong Kim

**Affiliations:** 1 Haeundae Bumin Hospital, Busan, Korea; 2 Department of Nursing, Pukyong National University, Busan, Korea

**Keywords:** Birth rate, Nurses, Reproductive behavior, Social support

## Abstract

**Purpose:**

South Korea’s rapid fertility decline, shaped by intertwined psychosocial and structural factors, is more severe than that observed in other Asia-Pacific and European countries. This study aimed to identify how determinants of childbearing intention vary among married, childless female Korean nurses using quantile regression.

**Methods:**

Married, childless female nurses younger than 45 years were recruited from seven cities in Korea. Participants (n=141) completed a self-administered survey between August and September 2024 on childbearing intention, health-related quality of life (HRQoL), self-actualization, social support, economic stability, and self-esteem. Data were analyzed using descriptive statistics, Pearson correlation coefficients, multiple linear regression, and quantile regression.

**Results:**

Participants reported moderate levels of childbearing intention and self-actualization, higher levels of HRQoL and social support, and moderate or higher levels of economic stability and self-esteem. In the multiple regression analysis, only age (B=1.67, *p*<.001) was a significant predictor of childbearing intention. Quantile regression revealed additional patterns: at the 60th percentile, age (B=1.89, *p*=.011), higher educational level (B=2.25, *p*=.040), and social support (B=1.06, *p*=.015) were significant, whereas at the 80th percentile, age (B=2.34, *p*=.001) and social support (B=1.41, *p*=.016) remained significant. These findings indicate that social support was strongly associated with childbearing intention among nurses with higher intention levels.

**Conclusion:**

Determinants of childbearing intention differed according to intention level, underscoring the value of moving beyond mean-based analyses. For married, childless female nurses, individualized interventions that enhance social support and account for age, educational level, and work-family context may be more effective than uniform, population-wide approaches in supporting childbearing intention.

## Introduction

The global total fertility rate (TFR) has steadily declined from approximately 5.0 in 1950 to 2.3 in 2021. In Asia-Pacific and European countries, including Japan, Singapore, and Germany, fertility rates of 1.0–1.6, which are below the replacement level, have already persisted for an extended period [1]. Although the pace varies, developing countries have also entered a phase of fertility decline, and a long-term downward convergence in global fertility rates has been observed [2]. Amid this global trend, South Korea’s TFR reached 0.78 in 2023, representing the lowest level in the world [1].

This decline in fertility rates is not merely a change in demographic indicators; it is also closely associated with structural shifts across individuals’ life-course transitions [3]. In South Korea, in particular, the traditional life course in which marriage and childbearing were naturally linked has gradually weakened since the 1980s, accompanied by delayed marriage, delayed childbearing, and a reduction in the number of children [4]. Furthermore, the proportion of married childless couples continues to increase, and this pattern is especially pronounced among highly educated women [5].

Amid these changes, childbearing can no longer be regarded as a natural life event. Rather, it should be understood as a planned decision that individuals make after weighing various conditions and resources [6,7]. In particular, childbearing intention is a key indicator of future childbearing behavior, reflecting cognitive and emotional states formed before childbirth [7]. Therefore, to understand and address declining fertility, it is necessary to examine systematically how childbearing intention is formed, rather than focusing solely on whether childbirth occurs.

Female nurses are an important population for research on childbearing intention because they are highly educated women who participate continuously in the labor force while simultaneously experiencing social expectations regarding marriage and childbirth and substantial occupational demands [8]. Previous studies have shown that nurses’ shift work, heavy workloads, emotional labor, and unpredictable schedules increase job stress and burnout. Beyond affecting job satisfaction or turnover intention alone, these factors may also function as structural constraints on major life decisions such as marriage, family formation, and childbearing [8,9]. In this context, married childless female nurses may perceive childbearing as a choice that could substantially affect career continuity and life direction. As a result, they are likely to undergo a more cautious and deliberate decision-making process [10].

Individual health status is an important condition in the formation of childbearing intention [11]. Health-related quality of life (HRQoL) encompasses perceived physical functioning, mental health, and social functioning [12] and has been reported to be closely associated with pregnancy preparation and long-term life planning among women of reproductive age [10]. In particular, health problems such as chronic fatigue, pain, and poor sleep quality may increase the psychological burden associated with pregnancy and childbirth [11,13]. Among nurses, whose work is characterized by high intensity, these health factors may play an even more important role in shaping childbearing intention [14]. In addition, childbearing is closely linked to individuals’ long-term life goals [15]. Self-actualization refers to the pursuit of professional achievement, meaning in life, and personal growth [16], and it may serve as a criterion by which individuals judge the place of childbearing in their lives [15,17]. When childbearing is perceived as compatible with life goals, childbearing intention may be strengthened [15]. Conversely, when it is perceived as conflicting with personal growth or career goals, childbearing intention may weaken [17].

Perceptions of resources available for childbearing and parenting also have an important influence on childbearing intention [6,8]. Social support refers to the physical, emotional, appraisal, and informational support provided by spouses, family members, and the workplace, and it shapes judgments about whether childbearing and parenting are realistically manageable [6,18]. In particular, among women exposed to high work intensity, social support has been reported to function as a key protective factor that alleviates uncertainty and burden related to childbearing [19].

Moreover, economic stability and self-esteem are factors that support the feasibility of childbearing plans and the intention to carry them out [20]. Structural conditions such as housing costs, income stability, and employment predictability have been reported to shape both the timing and feasibility of childbearing plans [21]. Self-esteem may also influence childbearing intention by helping individuals view their role performance and life control more positively, thereby functioning as a psychological resource for carrying out major life decisions [22].

Childbearing intention has been reported to deviate from a normal distribution even in large samples [6]. This distributional characteristic makes it difficult to satisfy the assumptions of traditional regression models that estimate effects based on the mean and suggests that influencing factors may operate differently across levels of childbearing intention. In contrast, quantile regression directly estimates conditional quantiles, making it possible to identify differential effects across intention levels. This approach may therefore be useful for informing policy and designing more precise nursing interventions. Accordingly, this study aimed to comprehensively analyze the effects of HRQoL, self-actualization, social support, economic stability, and self-esteem on first childbearing intention among married childless female nurses. In particular, by applying quantile regression, this study compared how the effects of these factors varied across low-, moderate-, and high-intention groups to characterize childbearing intention more precisely by level. Through this analysis, the study sought to examine empirically the formation of childbearing intention among female nurses in the context of ultra-low fertility and to provide a basis for tailored intervention strategies that reflect the characteristics of the nursing population.

The purpose of this study was to identify multidimensional factors affecting the level of childbearing intention among married childless female nurses. The specific objectives were as follows:

1) To identify participants’ general characteristics, HRQoL, self-actualization, social support, economic stability, self-esteem, and level of childbearing intention.

2) To analyze differences in the level of childbearing intention according to participants’ general characteristics.

3) To examine correlations between childbearing intention and HRQoL, self-actualization, social support, economic stability, and self-esteem.

4) To identify differential patterns in the effects of factors influencing childbearing intention across childbearing intention levels.

## Methods

**Ethics statement:** This study was approved by the Institutional Review Board of Pukyong National University (No. 1041386-202408-HR-114-02). Informed consent was obtained from the participants.

### Study design

This study employed a descriptive correlational design to analyze factors influencing childbearing intention among married childless female nurses. The study was reported in accordance with the STROBE (Strengthening the Reporting of Observational Studies in Epidemiology) guidelines (https://www.strobe-statement.org/).

### Participants

Participants were married childless female nurses aged 45 years or younger who were working in medical or non-medical institutions and who voluntarily agreed to participate. To focus on first childbearing intention, nurses who were currently pregnant were excluded. The minimum required sample size was calculated using G*Power ver. 3.1.9.7 (Heinrich Heine University Düsseldorf, Düsseldorf, Germany). Based on a medium effect size (Cohen’s d=0.5), a 95% confidence level, 80% statistical power, and 14 predictors, the minimum sample size was estimated to be 135. Allowing for a dropout rate of 10%, data were collected from 149 participants. After excluding three participants older than 45 years and five participants with missing values, data from 141 participants were included in the complete-case analysis.

### Measurements

All variables were measured using instruments with established reliability and validity, and permission to use the instruments was obtained from the original developers and translators. Mean scores were calculated for the main variables, which were then treated as continuous variables in the analysis.

#### Childbearing intention

Childbearing intention was measured using the prospective London Measure of Unplanned Pregnancy (pLMUP), which was translated into Korean by the researcher. The pLMUP is a modified version of the London Measure of Unplanned Pregnancy (LMUP), originally developed by Barrett et al. [23] to assess the planning and intentionality of pregnancy retrospectively and later adapted by Kavanaugh and Schwarz [24] to prospectively assess future pregnancy intention. The pLMUP consists of five items addressing contraceptive use since the last menstrual period, appropriateness of the timing of a pregnancy in the immediate future, pregnancy intention, discussion and agreement with a partner regarding the possibility of becoming pregnant, and health preparation for pregnancy since the last menstrual period. Each item is scored from 0 to 2, with higher total scores (possible range, 0–10) indicating higher levels of pregnancy planning and intention. Cronbach’s α was .92 in the original LMUP development study, .76 in the study by Ralph et al. [7] using the pLMUP, and .77 in the present study.

#### Health-related quality of life

HRQoL was measured using the Korean version [25] of the Medical Outcomes Study 36-item Short-Form Health Survey (SF-36), originally developed by Ware and Sherbourne [12]. The instrument consists of 36 items: 35 items across eight domains—physical functioning, role limitations due to physical health, bodily pain, general health, vitality, social functioning, role limitations due to emotional problems, and mental health—and one item assessing perceived health change compared with the previous year. The SF-36 uses different scoring systems across domains, and the item assessing perceived health change is not included in the total scoring. Physical functioning (10 items) is scored on a 3-point scale (1=limited a lot, 3=not limited at all). Role limitations due to physical health (four items) and role limitations due to emotional problems (three items) are scored on a 2-point scale (1=yes, 2=no). Bodily pain (two items), social functioning (two items), and general health (five items) are scored on a 5-point scale (1=excellent, 5=poor). Vitality (four items) and mental health (five items) are scored on a 6-point scale (1=all of the time, 6=none of the time). To ensure consistency in score direction, negatively worded items were reverse-coded. Raw scores for each domain were transformed to a 0 to 100 scale, summed, and then divided by the number of items in the corresponding domain. Higher transformed scores (possible range, 0–100) indicate a higher level of HRQoL. Cronbach’s α ranged from .78 to .93 across domains in the original study, from .51 to .85 in the Korean version, and was .91 in the present study.

#### Self-actualization

Self-actualization was measured using a scale developed by Min [26] to assess bureaucratic organizational characteristics and self-fulfillment. The instrument consists of 25 items across three domains: ability expression, ability development, and realization of higher goals. Each item was scored on a 5-point Likert scale (1=not at all, 5=very much so), and the total score was divided by the number of items to calculate a mean score (possible range, 1–5). Higher mean scores indicate higher levels of self-actualization. Cronbach’s α was .76 in the original study and .91 in the present study.

#### Social support

Social support was measured using the Korean version [27] of the Multidimensional Scale of Perceived Social Support, originally developed by Zimet et al. [28]. The instrument consists of 12 items assessing perceived social support from family, friends, and a significant other. Each item was scored on a 5-point Likert scale (1=very strongly disagree, 5=very strongly agree), and the total score was divided by the number of items to calculate a mean score (possible range, 1–5). Higher mean scores indicate higher levels of social support. Cronbach’s α was .88 in the original study, .89 in the Korean version, and .93 in the present study.

#### Economic stability

Economic stability was measured using an economic status scale developed by Park [29]. The scale consists of five items assessing satisfaction with average monthly income, economic independence, methods of covering living expenses, satisfaction with living expenses, and satisfaction with personal allowance. Each item was scored on a 5-point Likert scale (1=not at all, 5=very much so), and the total score was divided by the number of items to calculate a mean score (possible range, 1–5). Higher mean scores indicate better economic status. Cronbach’s α was .75 in the original study and .67 in the present study.

#### Self-esteem

Self-esteem was measured using the Korean version [30] of the Rosenberg Self-Esteem Scale, originally developed by Rosenberg [31]. The instrument consists of 10 items, including five positively worded items and five negatively worded items. Each item was scored on a 5-point Likert scale (1=strongly disagree, 5=strongly agree), and the negatively worded items were reverse-scored. The total score was divided by the number of items to calculate a mean score (possible range, 1–5). Higher mean scores indicate higher levels of self-esteem. Cronbach’s α was .92 in the original study, .85 in the Korean version, and .90 in the present study.

#### General characteristics

General characteristics included age, educational level, workplace, monthly household income, residential status, housing cost-to-income ratio, satisfaction with the husband’s participation in housework, and the presence of childcare providers [6,21,32].

### Data collection

Data were collected from August 19 to September 15, 2024, using convenience sampling. Participants were recruited from 14 tertiary and general hospitals located in Seoul, Busan, Dongtan, Pyeongchon, Yangsan, Geoje, and Cheonan, as well as from seven non-medical institutions, including public health centers, community service centers, schools, and firefighter training institutions. In medical institutions, questionnaires were distributed and collected with the cooperation of team leaders after permission had been obtained from the head of each department. Participants were fully informed about the purpose and procedures of the study through the participant information sheet and informed consent form provided on the first page of the questionnaire, and those who agreed to participate completed the survey voluntarily. In non-medical institutions, the researcher contacted each institution by telephone to explain the purpose and content of the study and then contacted eligible participants individually to confirm their willingness to participate. Written consent forms and questionnaires were mailed to those who agreed to participate. After written consent had been obtained, participants completed the questionnaires, which were later collected in person by the researcher. The survey required approximately 10 to 15 minutes to complete, and all participants who completed it received a small gift worth approximately Korean won (KRW) 5,000 as a token of appreciation.

### Data analysis

The collected data were analyzed using IBM SPSS Statistics ver. 29.0 (IBM Corp., Armonk, NY, USA) and StataNow/MP for Windows ver. 19.5 (StataCorp LLC, College Station, TX, USA). Levels of childbearing intention, HRQoL, self-actualization, social support, economic stability, and self-esteem were described using means and standard deviations. Participants’ general characteristics were summarized using frequencies, percentages, means, and standard deviations. Differences in childbearing intention according to general characteristics were analyzed using the independent t-test and one-way analysis of variance, and when significant differences were identified, the Scheffé post hoc test was conducted to examine between-group differences. Correlations between childbearing intention and the main variables were analyzed using Pearson correlation coefficients. Multiple regression analysis was performed to identify factors influencing childbearing intention. In this analysis, age and educational level, which showed significant differences in childbearing intention among the general characteristics, were included as control variables. In addition, HRQoL, self-actualization, social support, economic stability, and self-esteem, which showed significant correlations with childbearing intention, were included as the main independent variables. No fixed criteria exist for selecting quantiles in quantile regression, and quantiles may be divided into quartiles, quintiles, or deciles depending on the research purpose [27]. In this study, the dependent variable, childbearing intention score, was analyzed at the 20th, 40th, 60th, and 80th percentiles. These results were then compared with those of the multiple regression analysis to examine differences in influencing factors across levels of childbearing intention.

## Results

### Differences in childbearing intention according to the general characteristics of married childless female nurses

Table 1 presents differences in childbearing intention according to the participants’ general characteristics. The mean age of the participants was 32.52±3.88 years. Most held a bachelor’s degree (80.1%) and were employed in medical institutions (95.0%). The most common monthly household income was KRW 5 million to less than KRW 8 million (56.0%), and rental housing (53.9%) was slightly more common than home ownership (46.1%). The most common housing cost-to-income ratio was 10% to 30% (58.8%). Regarding satisfaction with husbands’ participation in housework, 70.2% of participants reported being at least satisfied. Slightly more participants reported not having childcare providers (53.9%) than having them (46.1%).

Among the participants’ general characteristics, age (F=9.50, *p*<.001) and educational level (t=–2.29, *p*=.023) showed statistically significant differences in childbearing intention. In particular, childbearing intention was significantly higher among participants aged 30 to 34 years. Participants with a master’s degree or higher also showed greater childbearing intention than those with a bachelor’s degree or lower.

### Correlations among childbearing intention, health-related quality of life, self-actualization, social support, economic stability, and self-esteem

Participants reported moderate levels of childbearing intention (5.72±2.78) and self-actualization (3.54±0.50), higher levels of HRQoL (77.71±10.46) and social support (4.24±0.62), and moderate or higher levels of economic stability (3.94±0.56) and self-esteem (3.86±0.60) (Table 2).

Correlation analysis showed that HRQoL (r=.20, *p*=.019), self-actualization (r=.23, *p*=.006), social support (r=.27, *p*=.001), economic stability (r=.17, *p*=.039), and self-esteem (r=.23, *p*=.005) were all significantly and weakly positively correlated with childbearing intention. Among the independent variables, the correlation between HRQoL and self-actualization was the lowest (r=.35, *p*<.001), whereas the correlation between self-esteem and self-actualization was the highest (r=.60, *p*<.001) (Table 3).

### Factors influencing childbearing intention by level

Assumption testing for the regression analysis showed tolerance values of 0.39–0.95 and variance inflation factor values of 1.05–2.55, indicating no evidence of multicollinearity. The Durbin-Watson statistic was 1.87, supporting the assumption of independence of residuals. Multiple regression analysis showed that only age was a significant predictor of childbearing intention, and the overall regression model was statistically significant (F=4.92, *p*<.001). The explanatory power of the model was 20.6%, and participants aged 30–34 years had significantly higher childbearing intention (B=1.67, *p*<.001).

In the quantile regression analysis, age (B=1.89, *p*=.011), social support (B=1.06, *p*=.015), and educational level (B=2.25, *p*=.040) were significant predictors at the 60th percentile, whereas age (B=2.34, *p*=.001) and social support (B=1.41, *p*=.016) were significant at the 80th percentile (Table 4).

## Discussion

This study is meaningful in that it applied quantile regression to analyze factors influencing childbearing intention among married childless female nurses and to identify differences across levels of childbearing intention. Although multiple regression analysis identified age as the only significant predictor of childbearing intention, quantile regression showed that educational level and social support emerged as additional factors in the groups with moderate to high levels of childbearing intention. Notably, in the 80th percentile group, social support was identified as the strongest predictor of childbearing intention. These findings provide three major implications centered on the importance of age, educational level, and social support in the formation of childbearing intention.

Age is closely related to the biological reproductive period. The findings of Safdari-Dehcheshmeh et al. [3], which showed that most nurses younger than 35 years reported childbearing intention, are consistent with the present finding that nurses aged 30 to 34 years had higher childbearing intention. This suggests that, when biological health and socioeconomic stability are considered together, women in their early thirties may perceive themselves as sufficiently prepared in terms of health and finances, resulting in higher childbearing intention. Whereas previous studies have largely emphasized age as a risk factor for declining childbearing intention and delayed childbearing [3,6], the present findings suggest that, among nurses, age may become a factor that strengthens childbearing intention after a certain point. Therefore, targeted interventions such as tailored health management programs, preconception care education, and psychosocial counseling may be needed for women aged 30 to 34 years. Because women in this age group face the dual demands of career stability and preparation for childbirth, institutional support, including a childbearing-friendly work environment and expanded flexible work arrangements, as suggested by Geng et al. [8], may increase the likelihood that childbearing intention is translated into actual childbirth.

Previous studies have often interpreted higher educational level as a negative factor in childbearing, based on findings that women with higher education tend to delay marriage and childbirth or have fewer children [33,34]. However, recent Korean studies have suggested that the relationship between educational level and childbearing intention is intertwined with socioeconomic status, marital status, and occupational characteristics, making it difficult to interpret as a simple inverse relationship [34,35]. Kim and Yi [6], in a systematic review and meta-analysis, reported that educational level had a significant positive association with childbearing intention. Similarly, Lee and Zeman [5], in a cohort study of Korean women, found that women with lower educational levels were more likely to remain childless. In the present study, nurses with a master’s degree or higher showed significantly greater childbearing intention at the moderate level (60th percentile) than those with a bachelor’s degree. This finding suggests that, among highly educated nurses with at least a moderate level of childbearing intention, educational attainment may function as a resource that supports economic and occupational stability as well as future career planning [9,15]. In other words, the general tendency for higher education to be associated with avoidance of childbearing may be modified in the nursing population according to job stability, organizational career pathways, and levels of social support [8,33]. However, this study did not identify the specific professional or organizational characteristics underlying these differences among highly educated nurses. Therefore, future research should examine the heterogeneity within this group and explore more precisely the pathways through which educational level influences the formation of childbearing intention.

The finding that social support influenced childbearing intention in this study is consistent with numerous empirical studies. Yoon [18] reported that spousal support for housework and childcare, as well as caregiving by co-residing parents or parents-in-law, significantly increased the likelihood of having a second child, suggesting that family-level social support is closely associated with subsequent childbearing behavior. Previous studies have generally identified social support as a protective factor with an average effect among married women or couples with children [32,36]. In the present study, however, social support was a significant predictor only in the groups with moderate to high levels of childbearing intention, and its effect was greatest in the 80th percentile group. This suggests that, among nurses facing substantial work-family conflict, social support may do more than increase childbearing intention overall; it may also serve as a key condition that facilitates the translation of positive childbearing intention into actual behavior among those already considering childbearing favorably. In light of these findings, social support should be considered as a targeted intervention that links established childbearing intention to perceived feasibility. Strategies such as strengthening emotional bonds within families, providing childbirth and parenting education for spouses and family members, and establishing peer-based support networks in the workplace may enhance psychological security and perceived feasibility among nurses with at least a moderate level of childbearing intention.

In this study, HRQoL, self-actualization, economic stability, and self-esteem showed significant weak correlations with childbearing intention. However, these variables did not emerge as statistically significant predictors in the quantile regression analyses across childbearing intention levels. These findings may be interpreted in several ways.

HRQoL and economic stability have been reported to function as basic prerequisites in childbearing decision-making among women of reproductive age [6]. However, among the married childless female nurses in this study, these factors may already have been satisfied above a certain threshold, thereby limiting variability. As a result, the effects of HRQoL and economic stability on childbearing intention may not have been sufficiently reflected in between-group differences. Future research should include women from a wider range of occupations and economic backgrounds to increase variability in these factors and to clarify their roles in shaping childbearing intention.

In this study, self-actualization was not significantly associated with childbearing intention. This finding suggests that childbearing decisions among professional women are shaped not simply by the pursuit of self-actualization but by the interaction of more complex psychosocial and structural factors [17,37]. In particular, the relationship between career orientation and childbearing intention has yielded inconsistent findings in previous studies. Simoni et al. [17] reported that career-oriented women tended to view childbearing more as a planned decision, but they also tended to delay actual childbirth. Zhen et al. [19] further suggested that a stronger emphasis on career may intensify conflict with family roles and thereby reduce childbearing intention. In contrast, Geng et al. [8] reported that “decent work,” which emphasizes the meaning and value of work, mediated the relationship between work-life balance and childbearing intention. This suggests that childbearing intention may increase when stable employment conditions and work-life balance are secured. Therefore, whether the pursuit of professional achievement conflicts with or complements childbearing intention remains to be clarified. Future research should use qualitative or mixed-methods approaches to provide a more in-depth understanding of these dynamics within the context of women’s lived experiences and multilayered conditions.

The finding that self-esteem did not significantly influence childbearing intention is consistent with some previous studies reporting that self-esteem does not directly explain reproductive behaviors such as pregnancy and childbirth [22]. Self-esteem reflects individuals’ overall self-evaluation [31], whereas childbearing intention is a decision strongly shaped by structural factors such as career sustainability, work-family balance, and institutional support [18]. Accordingly, general psychological characteristics such as self-esteem may have limited value in directly explaining childbearing intention, and the present findings may reflect this conceptual distinction. In contrast, job-related self-efficacy has been reported to moderate work-family conflict [38]. Such characteristics may indirectly influence the formation of childbearing intention by shaping perceived feasibility and burden related to balancing childbearing and parenting. Therefore, future research should examine more systematically the complex relationships and indirect effects among variables by incorporating job-related psychological factors such as job-related self-efficacy and stress-coping ability.

This study has several limitations. Many previous studies assessed childbearing intention using dichotomous nominal variables such as “yes” or “no,” making it difficult to capture its continuous nature. To address this limitation, the present study used a translated version of the pLMUP, a tool developed to prospectively assess pregnancy planning and intention, to evaluate childbearing intention at a more granular level. However, because the pLMUP was originally developed to assess the relationship between pregnancy intention and obstetric outcomes, the validity and reliability of using it to measure childbearing intention during the preconception period have not been fully established. Therefore, future research is needed both to validate the pLMUP for assessing future childbearing intention and to develop new instruments optimized for the continuous assessment of future childbearing intention. In addition, because this study included only married childless female nurses, the findings may not be generalizable to the broader female population or to other occupational groups. Although the sample size was sufficient for regression analysis, it may be insufficient for generalizing factors influencing childbearing intention across levels. Therefore, future large-scale studies including more diverse regions and occupations are needed.

Despite these limitations, this study provides a basis for a more multidimensional understanding of the formation of childbearing intention among nurses by comprehensively analyzing factors influencing first childbearing intention, including HRQoL, self-actualization, social support, economic stability, and self-esteem, among married childless female nurses. In addition, by using quantile regression, this study empirically examined differences in influencing factors across levels of childbearing intention and showed that the effects of the same factors may vary among groups with low, moderate, and high levels of childbearing intention. In doing so, the study helps address the limitations of previous research that interpreted childbearing intention solely on the basis of a single mean value. Furthermore, the findings may serve as a basis for tailored intervention strategies and low-fertility policy development that reflect the distinct professional and social context of female nurses in an era of ultra-low fertility, including strengthening social support, improving the work environment, and providing health and psychological support resources.

In conclusion, the analysis of factors influencing childbearing intention among married childless female nurses showed that age was the only significant predictor in the multiple regression analysis, whereas educational level and social support emerged as additional influential factors in the groups with moderate to high levels of childbearing intention in the quantile regression analysis. Notably, social support was the strongest factor associated with childbearing intention in the 80th percentile group. In contrast, although HRQoL, economic stability, self-actualization, and self-esteem were correlated with childbearing intention, they did not emerge as significant predictors in the level-specific analyses. These findings suggest that childbearing intention may be shaped more strongly by the work environment, social resources, and structural conditions than by general psychological characteristics. Overall, the findings indicate that nurses’ childbearing intention is not adequately explained by a single average effect, but instead reflects a multilayered decision-making process in which different factors operate selectively according to the level of childbearing intention.

Based on these findings, several recommendations can be made. From the perspective of nursing research, childbearing intention should be understood not as a phenomenon explained by a single mean value, but as a multidimensional decision-making process in which different determinants operate selectively according to intention level. Because this study identified differences in influencing factors across childbearing intention levels, future research should examine the heterogeneity of these factors more precisely in populations with diverse occupations and marital statuses. From the perspective of nursing practice, targeted interventions for nurses should focus particularly on strengthening social support and improving the work environment among those with at least a moderate level of childbearing intention.

## Figures and Tables

**Table 1.** Childbearing intention by participants’ general characteristics (N=141)

Characteristics	Categories	n (%)	Mean±SD	t/F (*p)*^†^
Age (year)			32.52±3.88	
	23–29^a^	33 (23.4)	4.45±2.33	9.50 (<.001) a, c<b
	30–34^b^	66 (46.8)	6.71±2.70	
	35–44^c^	42 (29.8)	5.17±2.70	
Educational level	≤Bachelor’s	113 (80.1)	5.46±2.77	–2.29 (.023)
	≥Master’s	28 (19.9)	6.79±2.60	
Workplace	Clinical	134 (95.0)	5.79±2.80	1.27 (.207)
	Non-clinical setting	7 (5.0)	4.43±1.99	
Monthly household income (KRW)	<5 million	14 (9.9)	5.00±2.75	0.54 (.586)
	5 to <8 million	79 (56.0)	5.77±2.81	
	≥8 million	48 (34.1)	5.85±2.77	
Residential status	Owner-occupied	65 (46.1)	5.97±2.81	0.97 (.333)
	Rented	76 (53.9)	5.51±2.75	
Housing cost-to-income ratio	<10%	29 (20.6)	5.48±2.67	0.15 (.865)
	10%–30%	83 (58.8)	5.81±2.70	
	>30%	29 (20.6)	5.72±3.16	
Satisfaction with husband’s housework	Less than satisfied	42 (29.8)	5.19±2.67	–1.49 (.138)
	Satisfied or very satisfied	99 (70.2)	5.95±2.80	
Childcare provider	Yes	65 (46.1)	5.63±2.59	0.37 (.716)
	No	76 (53.9)	5.80±2.94	

KRW: Korean won (1 million KRW is approximately 700 US dollars).

† Scheffé test.

**Table 2.** Main variables (N=141)

Variables	Possible range	Mean±SD (range)
Childbearing intention	0–10	5.72±2.78 (0–10)
Health-related quality of life	0–100	77.71±10.46 (29.58–97.50)
Self-actualization	1–5	3.54±0.50 (2.32–5.00)
Social support	1–5	4.24±0.62 (2.25–5.00)
Economic stability	1–5	3.94±0.56 (2.20–5.00)
Self-esteem	1–5	3.86±0.60 (1.20–5.00)

**Table 3.** Correlations among health-related quality of life, self-actualization, social support, economic stability, self-esteem, and childbearing intention (N=141)

Variables	r (*p*)				
	1	2	3	4	5
1. Childbearing intention	1				
2. Health-related quality of life	.20 (.019)	1			
3. Self-actualization	.23 (.006)	.35 (<.001)	1		
4. Social support	.27 (.001)	.37 (<.001)	.51 (<.001)	1	
5. Economic stability	.17 (.039)	.36 (<.001)	.46 (<.001)	.48 (<.001)	1
6. Self-esteem	.23 (.005)	.60 (<.001)	.60 (<.001)	.58 (<.001)	.45 (<.001)

**Table 4.** Factors influencing childbearing intention by intention level (N=141)

Variables	B (*p*)				
	Quantile regression				MLR
	0.2	0.4	0.6	0.8	
Age (30–34 years)	1.24 (.058)	1.49 (.076)	1.89 (.011)	2.34 (.001)	1.67 (<.001)
Educational level (≥Master’s)	1.49 (.105)	0.92 (.362)	2.25 (.040)	1.82 (.057)	1.15 (.050)
Health-related quality of life	0.01 (.856)	0.03 (.475)	0.30 (.336)	0.01 (.876)	0.02 (.554)
Self-actualization	0.80 (.336)	0.74 (.490)	–0.39 (.164)	–0.18 (.862)	0.41 (.479)
Social support	–0.32 (.661)	0.52 (.589)	1.06 (.015)	1.41 (.016)	0.82 (.080)
Economic stability	1.00 (.255)	–0.14 (.883)	1.82 (.687)	0.67 (.316)	0.19 (.687)
Self-esteem	0.11 (.893)	–0.28 (.769)	–0.39 (.164)	–0.71 (.349)	–0.24 (.675)

Dummy variable for age: 30–34 years=1, otherwise (<30 or ≥35)=0. MLR: Multiple linear regression.
